# Epidemic Influenza: A Study in Comparative Statistics

**Published:** 1893-06

**Authors:** 


*Epidemic Influenza: a Study in Comparative Statistics.* By F. A. DIXEY, D.M. Oxford: At the Clarendon Press. 1892.

In this book of elaborate statistics, which appear to be most carefully compiled and tabulated, Dr. Dixey has not only compared the older epidemics of 1847–1848 with the more recent outbreaks from 1889 onwards; but has dealt in detail with the 1891-92 influenza in London, and with the course of the same epidemic in Paris, Berlin, and other cities. A large portion of the book consists of tables and graphic charts representing with great clearness the results of Dr. Dixey's study. The volume should prove useful to the statistician and the student of influenza.

